# A comparison of the Macintosh laryngoscope, McGrath video laryngoscope, and Pentax Airway Scope in paediatric nasotracheal intubation

**DOI:** 10.1038/s41598-018-35857-8

**Published:** 2018-11-26

**Authors:** Ji Young Yoo, Yun Jeong Chae, Young Bok Lee, Sujin Kim, Jaemoon Lee, Dae Hee Kim

**Affiliations:** 1 Department of Anesthesiology and Pain Medicine, Ajou University School of Medicine, Suwon, Republic of Korea; 2 Department of Anesthesiology and Pain Medicine, Yonsei University, Wonju College of Medicine, Wonju, Republic of Korea

## Abstract

We evaluated the performance of the McGrath video laryngoscope and Pentax Airway Scope in comparison with the Macintosh laryngoscope for nasotracheal intubation in paediatric patients. For this, 108 patients were enrolled in an open-label, randomized controlled trial. Patients were randomly allocated to one of three groups based on use of the Macintosh laryngoscope, McGrath video laryngoscope, or Pentax Airway Scope. Time to intubation, the intubation difficulty, and the quality of navigation were compared among groups. The median nasotracheal intubation time [interquartile range] in the Macintosh group (33.5 [28.3–39.8] s) was significantly shorter than those of the McGrath (39.0 [32.0–43.0] s) and Pentax groups (43.0 [35.0–52.0] s). The difficulty of nasotracheal intubation was similar among all groups. When navigating and aligning the tube from the oropharynx into the glottic inlet, the cuff inflation method was required in significantly fewer patients for the Macintosh group (11.1%) than for the McGrath (48.6%) and Pentax (51.4%) groups. Thus, compared to the McGrath video laryngoscope and Pentax Airway Scope, the Macintosh laryngoscope allowed shorter nasotracheal intubation times and better facilitated tracheal navigation, requiring less use of the cuff inflation method to navigate the tracheal tube into the glottic inlet.

## Introduction

Increasing evidence suggests that use of a video laryngoscope in nasotracheal intubations can offer several advantages over a direct laryngoscope^1–3^. Video laryngoscopes not only provide a clear view of the glottis, but also facilitate navigation of the tracheal tube into the glottic orifice^1,3–6^. Furthermore, unlike conventional laryngoscopes, which provide direct line-of-sight, video laryngoscopes provide a non-line-of-sight view through a screen display, providing greater visibility when advancing the tracheal tube into the trachea^2,5,6^. This decreases distortion of the oropharyngeal structure, and minimizes the need for Magill forceps to insert the tracheal tube and reduces the time needed to intubate. Accordingly, shorter intubation times, ease of intubation, less use of optimization manoeuvres, and higher success rates of intubation associated with the use of a video laryngoscope have frequently been reported^2,5–8^.

It is important to note that the studies mentioned here primarily investigated nasotracheal intubation in adult patients. The airways of paediatric patients are anatomically different to adults, for example, in the cephalad larynx and posterior angled trachea^9,10^. Therefore, the expected advantages of video laryngoscopes in adults are not necessarily manifested in paediatric patients, and very few studies supporting this have been reported^11^. According to a previous study on paediatric nasal intubation, the GlideScope video laryngoscope showed similar intubation performance with a direct laryngoscope in terms of the need for use of Magill forceps and total intubation times^11^. The GlideScope is an angulated blade among three categories of video laryngoscope: Macintosh-shaped blade, angulated blade (anatomically-shaped without tube guide), and channelled blade (anatomically-shaped with tube guide)^1,12^. Currently, no comparative studies have been performed using the Macintosh-shaped and channelled blade in paediatric patients.

The objective of this investigation was to compare the intubation time, difficulty of nasotracheal intubation, and quality of navigation at each stage of tube insertion of the Macintosh laryngoscope, McGrath video laryngoscope, and Pentax Airway Scope for nasotracheal intubation in paediatric patients.

## Results

**Figure 1:**
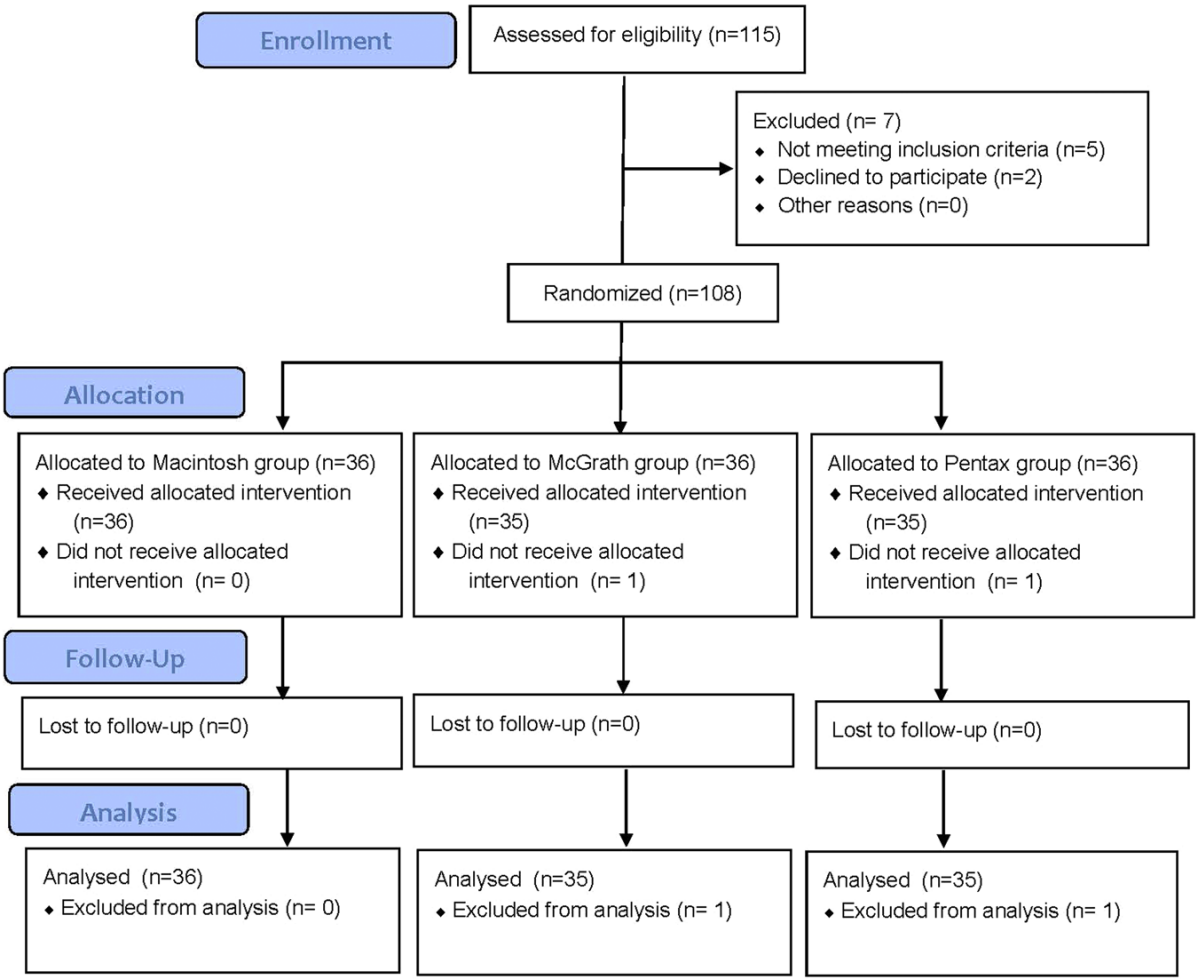
CONSORT flow diagram of recruitment and assessment of study participants.

Out of 115 patients screened, 108 were selected and assigned randomly into one of three groups corresponding to each laryngoscope. The participant flow diagram for the study is presented in Fig. 1. Nasotracheal intubation was completed within 120 s in all patients, but two patients (from the McGrath and Pentax groups) were categorized as a failed intubation because they were intubated with a Macintosh laryngoscope instead of the assigned device.

All three groups were similar with respect to age, sex, weight, height, and American Society of Anesthesiologists physical status (Table 1). Glottic visualization using the modified Cormack and Lehane grading system was significantly clearer in McGrath group and Pentax groups than in the Macintosh group (P < 0.001).

**Table 1:** Patient characteristics and airway assessment.

Variables	Group Macintosh(n = 36)	Group McGrath(n = 35)	Group Pentax(n = 35)
Age (years)	7 (6–8)	7 (6–8)	7 (5–8)
Sex (male/female)	28/8	27/9	25/11
Weight (kg)	25 (22–30)	25 (22–34)	25 (19–32)
Height (cm)	125 (118–132)	126 (118–134)	124 (115–130)
ASA grade (I/II)	35/1	34/1	34/1
Nostril used (right/left)	30/6	31/4	31/4
MC-L grade (I/IIa/IIb/III/IV)	15/12/6/3/0	34/1/0/0/0*	31/4/0/0/0*

Values are presented as median (interquartile range), or number of patients. ASA, American Society of Anesthesiologists; MC-L, modified Cormack-Lehane. *P < 0.05 compared to data of group Macintosh.

The intubation times of each group varied significantly (P = 0.001); the median time taken for intubation (interquartile range) of the Macintosh, McGrath, and Pentax groups were 33.5 s (28.3–39.8 s), 39.0 s (32.0–43.0 s), and 43.0 s (35.0–52.0 s), respectively (Table 2). The Hodges-Lehman median difference between the Macintosh group and the McGrath and Pentax groups was −5.0 s (95% confidence interval [CI]; −9.0 to −1.0 s, P = 0.016) and −10.0 s (95% CI; −15.0 to −5.0 s, P < 0.001), respectively. There was no significant difference between the McGrath and Pentax groups (median difference; −5.0 s, 95% CI; −11.0 to 0.0 s, P = 0.061).

**Table 2:** Intubation data.

	Group Macintosh (n = 36)	Group McGrath (n = 35)	Group Pentax (n = 35)	P-value
Time to intubation (sec)	33.5 (28.3–39.8)	39.0 (32.0–43.0)*	43.0 (35.0–52.0)*	0.001
NIDS score	1 (0–2)	1 (0–1)	2 (1–2)	0.633
NRS (0–10)	1 (0–2)	1 (0–1.8)	2 (1–2)	0.144
**Quality of navigation**				
Nose to oropharynx (1/2/3)	19/10/7	22/9/4	25/6/4	0.572
Oropharynx to glottic inlet (1/2/3)	32/4/0	18/17/0*	17/18/0*	<0.001
Glottic inlet to trachea (1/2/3)	6/29/1	7/27/1	24/11/0*	<0.001
Epistaxis (1/2/3/4)	24/9/3/0	26/9/0/0	29/6/0/0	0.203

Values are presented as median (interquartile range), or number of patients. NIDS, nasal intubation difficulty score; NRS, numeric-rating scale. *P < 0.05 compared to data of group Macintosh.

The difficulty of nasotracheal intubation using the modified nasal intubation difficulty score (NIDS) and numeric rating scale (NRS) did not differ significantly between the three groups (Table 2). In terms of the quality of navigation, the insertion of tube into the nostril and to the oropharynx was similar between the three groups, but there were significant differences in the alignment of the tube from the oropharynx to the glottic inlet, and the advancement of the tube from the glottic inlet to the trachea (Table 2). As a percentage of patients, during the alignment of the tube from the oropharynx to the glottic inlet, the cuff inflation method was more frequently used in the McGrath (48.6%) and Pentax groups (51.4%) than in the Macintosh group (11.1%). During the advancement of the tube from the glottic inlet to the trachea, the tube-rotation method was more frequently used in the Macintosh (80.6%) and McGrath groups (77.1%) than in the Pentax group (31.4%). There were no differences in the incidence of epistaxis among the groups.

## Discussion

In this study, the application of three laryngoscopes in paediatric nasotracheal intubation was investigated in a trial involving 108 patients between ages 1 and 10 years. We demonstrated that the Macintosh laryngoscope required shorter intubation times than did the McGrath video laryngoscope or Pentax Airway Scope. In addition, during the alignment of the tube from the oropharynx to the glottic inlet, the cuff inflation method was required less frequently in the Macintosh group than in the McGrath and Pentax groups.

Our findings are in contrary to that of previous reports in adults. In adult nasotracheal intubation, video laryngoscopes have frequently been reported to display significant advantages over the Macintosh laryngoscope^1–3^. Video laryngoscopes have been shown to provide improved glottic exposure and non-line-of-sight view, thereby allowing quick visualization of the glottic inlet and easy navigation of the tracheal tube through a less distorted anterior airway^2,3,6,7^. Thus, many reports have concluded these scopes as superior tools with regard to success rates and/or intubation time^4–8^. In studies using Magill forceps as a navigation method, the incidence of the use of Magill forceps with Macintosh laryngoscopes ranged between 34% and 49%, while with video laryngoscopes, the incidence ranged between 0% and 6%^6,7^. In patients aged 8–18 years, the cuff inflation method and Magill forceps were used in 6.7% to 60% of patients when using the Macintosh laryngoscope and in 6.7% to 17% of patients using the C-MAC video laryngoscope^13^. Therefore, most existing evidence suggests that compared to Macintosh laryngoscopies, video laryngoscopies require less use of Magill forceps and are associated with reduced intubation times. On the other hand, our study, which focused on paediatric patients, suggests that Magill forceps are not required for tube alignment from the oropharynx to the glottic inlet for any of the laryngoscopes investigated. The cuff inflation method, when used as needed, was sufficient for aligning the tube into the glottic inlet. Moreover, the cuff inflation method was used for only 4 patients (11.1%) in the Macintosh group, in contrast to 17 (48.6%) and 18 (51.4%) patients in the McGrath and Pentax groups, respectively. The cuff inflation method takes time to inflate the cuff and lift the tracheal tube from the posterior pharyngeal wall, as well as requiring manipulation of the tracheal tube tip into the glottic inlet, and deflating of the ballooned cuff. In a previous study, the intubation time in patients requiring the cuff inflation method was reported to be 7.2 s longer on average than that in patients where inflation was not used^14^. Therefore, in this study, variation in the need for cuff inflation contributed to shorter intubation times in the Macintosh group in comparison to the times in the McGrath and Pentax groups.

This study is unique compared with previous studies because of the differences in the anatomy of paediatric airways compared to the anatomy of adult airways, as well as the use of a reinforced tube. The paediatric laryngeal structure differs from adults, including a relatively larger tongue and a more cephalad larynx, which may increase the complexity of nasotracheal intubation in children^11^. In particular, the more cephalad larynx and posterior angled trachea of paediatric airways is thought to cause difficulty when advancing the tube into the trachea^10^. Furthermore, the use of a reinforced tube, which is softer than a normal polyvinyl chloride preformed tube, has been shown to adversely affect nasotracheal alignment of the tube with the glottic inlet owing to its tendency to move along the posterior pharyngeal wall^15,16^. This, however, was not observed with the use of the reinforced tube in this study, with 88.9% of the Macintosh group being scored grade 1, which indicates easy alignment of the tube into the glottic inlet without the use of the cuff inflation or Magill forceps. As expected, the McGrath and Pentax groups received superior grades on the modified Cormack and Lehane system. However, the majority of patients in the Macintosh group were within grades 1-2, with some becoming grade 1-2 with the application of backwards upwards rightward pressure, suggesting that visualization of the glottic inlet was as efficient and clear as that for the video laryngoscopes.

In addition, the thermally softened reinforced tube, when used in the Macintosh group, showed good performance, contrary to what was expected. In our experience, the tube maintained a gentle curvature that was less prominent than that of the preformed tube, and it did not touch the posterior pharyngeal wall. The gap between the tube tip and the posterior pharyngeal wall formed by this slight curvature was enough to align the tube tip with the glottic inlet using a to-and-fro or right/left turning movement by right-hand manipulation alone. On the other hand, in a previous investigation of paediatric nasotracheal intubation in patients less than 10 years of age using a preformed tube, Magill forceps were required for most children when using either the Macintosh or GlideScope video laryngoscopes^11^. We assumed, cautiously, that the small distance between the posterior laryngeal wall and the glottic inlet in the paediatric anatomy was similar to the gap between the tube tip and the posterior pharyngeal wall formed by this curvature, so the gentle curvature of a thermally softened reinforced tube may in fact be advantageous for the alignment of the tube into the glottic inlet than the curvature of a preformed tube. However, this effect may also be attributed to differences in experience with and preference of alignment methods of the anaesthesiologist. We therefore propose that further investigation of the effect of curvature between preformed and reinforced tubes on the difficulty of the alignment in paediatric patients is required.

Compared to the Macintosh group, the McGrath and Pentax groups required the cuff inflation method more frequently. This was presumably caused by differences between the direct and indirect view of the investigated laryngoscopes. Indirect view of video laryngoscope is advantageous to visualization of the glottic inlet, but the field around the glottic inlet on the video display is narrower compared to the direct view of Macintosh laryngoscope^17,18^. Our results indicate that the direct view provided by the Macintosh laryngoscope of the areas between the tracheal tube and the glottic inlet is important for the alignment of the tracheal tube tip into the glottic inlet via manipulation and movement of the tracheal tube with the right hand. On the other hand, the indirect view provided by the video laryngoscopes could not maintain visualization of the tip of the tracheal tube due to its narrow field of view. Therefore, manipulation of the tube was less straightforward, and the cuff inflation method was used instead for the video laryngoscopes.

During advancement of the tube into the trachea after alignment with the glottic inlet, the reinforced tube often encountered resistance. The incidence of resistance was 83.3%, 80.0%, and 31.4% in the Macintosh, McGrath, and Pentax groups, respectively, compared to 20% in a previous paediatric study in which a preformed tube was used^11^. Therefore, the reinforced tube more frequently required the tube-rotation method to advance in comparison to a preformed tube. However, this resistance was easily overcome by employing clockwise rotation of the tracheal tube, except in only three patients among all groups. Rotation of the tube changes the direction of its natural curvature^19^, which is sufficient to overcome resistance due to adherence of the tube tip to the anterior surface of the glottic inlet. Importantly, it is easier to change the direction of a flexible, softened reinforced tube than a polyvinyl chloride tube^19^, so the process of rotation was simple and quick to perform in this study. However, caution should be taken when rotating the tip: the degree of rotation at the proximal end of the tracheal tube may be over 180°, as proximal rotation does not always result in equivalent rotation of the distal tip of the tube^20^. Three patients in this study required the use of Magill forceps for advancing the tube (1 from the Macintosh group and 2 from the McGrath group). The low incidence of resistance during advancement in the Pentax group suggests that non-line-of-sight view of Pentax Airway Scope minimized the distortion of the oropharyngeal structure^2,5,6^, providing a smoother advancement than did the Macintosh laryngoscope and Macintosh-type blade of the McGrath video laryngoscope.

In paediatric patients, longer intubation times are a significant drawback, as the allowed duration of intubation is shorter than that of adults^21^. Despite requiring longer intubation times than the Macintosh group, the upper margin of the CI in the McGrath and Pentax groups (95% CI: 36.0–46.2 s and 40.8–50.8 s, respectively) was less than 60 s. This is shorter than that in a previous paediatric study using direct laryngoscopes and GlideScope video laryngoscopes with Magill forceps^11^. We therefore believe that these times are still within a clinically acceptable range. Moreover, the difficulty of nasotracheal intubation using the modified NIDS and NRS was similar among the three groups, suggesting McGrath video laryngoscopes and Pentax Airway Scopes are also feasible options in paediatric patients. Although the intubation times of the video laryngoscopes were longer than that of Macintosh laryngoscope, there may be cases where use of a video laryngoscope is necessary due to a poor view of the glottis by direct laryngoscopy.

Two cases were categorized as failed intubations because they were intubated with a Macintosh laryngoscope instead of the assigned video laryngoscope. In a patient from the McGrath group, advancement of the tube from glottic inlet to trachea failed with the tube-rotation method, and we encountered difficulties inserting Magill forceps into the oropharynx due to limited room. Thus, the Macintosh laryngoscope and Magill forceps were used at the discretion of the anaesthesiologist. In a patient from the Pentax group, blood pooling in the oropharynx obstructed the view, so intubation was performed with a Macintosh laryngoscope. Regardless of the type of video laryngoscope, any blood or secretions can obstruct the indirect view, which can complicate intubation^22^. Furthermore, the limited room available for insertion of Magill forceps when using video laryngoscope can be problematic, especially in nasotracheal intubations^11^.

Some limitations are associated with this study. First, the anaesthesiologist performing intubations could not be blinded to the group allocation. Second, all nasotracheal intubations were performed by a single experienced anaesthesiologist, so the generalizability of our findings to less experienced users might be limited; intubation may take longer for an inexperienced user. Third, all nasal intubations were performed in normal airways; in difficult airways, the results may differ.

In conclusion, in contrast to findings based on nasotracheal intubation in adult patients, performing nasotracheal intubation in paediatric patients with the Macintosh laryngoscope resulted in shorter intubation times than did both the McGrath video laryngoscope and Pentax Airway Scope. In addition, the Macintosh laryngoscope required less frequent use of the cuff inflation method to navigate the tracheal tube into the glottic inlet than did the McGrath video laryngoscope and Pentax Airway Scope. While video laryngoscopies can offer significant advantages, especially in adult patients or where there is poor view of the glottis, a direct laryngoscope seems to allow for more efficient nasotracheal intubation in paediatric patients.

## Methods

The protocol of this prospective, randomized, open-label, single-centre, three parallel group, controlled study was approved by the Institutional Review Board of Ajou University Hospital, Suwon, South Korea (approval number: AJIRB-MED-OBS-16-145) and conducted in accordance with the tenets of the Declaration of Helsinki. The study was registered in ClinicalTrials.gov on 11 July 2016 (registration number: NCT02828631).

### Patient selection

Patients with American Society of Anesthesiologists physical status of I or II, aged 1 to 10 years, and undergoing dental surgery under general anaesthesia were eligible to participate in this study. Children with any anatomical abnormality of the face, coagulation disorders, a history of a difficult airway, asthma, or upper respiratory infection within 14 days of the study were excluded. Written informed consent was obtained from all parents and the children themselves over six years old according to advice of the Institutional Review Board. Between June 2016 and October 2017, 108 children at the Ajou University Hospital were enrolled and randomly assigned to one of three groups in which the Macintosh laryngoscope, McGrath video laryngoscope, or Pentax Airway Scope was used. An independent colleague not involved with this research conducted the random group assignment using randomization software. Immediately before inducing anaesthesia, a sealed opaque envelope containing a number indicating the selected laryngoscope was provided to the anaesthesiologist and opened in the operating room.

### Anaesthesia

No patients were pre-medicated before surgery. A peripheral intravenous catheter was inserted in the ward or waiting room, and patients entered the operating room with a parent present. Standard monitoring including electrocardiography, non-invasive monitoring of blood pressure, pulse oximetry, and capnography were performed throughout the procedure. The patient was placed in the supine position and their head and neck was supported on a pillow for sniffing position to align the tragus of the ear with the patient’s sternal notch. This occurred before anaesthesia induction in cooperative patients and after anaesthesia induction for uncooperative patients. Patients were asked which side of the nose was easier to breathe through and the corresponding nostril was selected for intubation. If both nostrils were equally easy to breathe through or the patient was uncooperative, the right nostril was selected. Following pre-oxygenation, general anaesthesia was induced with thiopental sodium (5 mg/kg) and fentanyl (1 µg/kg) to induce loss of consciousness. After induction of anaesthesia, manual ventilation by mask was carried out with sevoflurane (end-tidal concentration: 2–3%) in oxygen, and rocuronium (0.6 mg/kg) was injected. Two minutes after the injection, nasotracheal intubation was performed using one of the assigned devices by an anaesthesiologist (Y.J.C.) experienced in the usage of all three devices.

The Lo-Contour cuffed reinforced tube (Mallinckrodt^TM^, Covidien, Ireland) was used for intubation. After softening the tube by immersion in heated sterile water for 5 min, it was coated with a water-soluble lubricant immediately prior to insertion. Suitable tracheal tube diameters were calculated for each patient according to the Motoyama formula (internal diameter, mm = [age/4] + 3.5)^23^.

### Measured parameters

The time taken for intubation was measured as the time between entry of the nasotracheal tube into a nostril and appearance of a carbon dioxide wave on the monitor. A failed intubation occurred when nasotracheal intubation could not be achieved within 120 s or with the use of a different modality. At any point, if the oxygen saturation decreased below 95%, the intubation attempt was terminated; manual ventilation resumed and it was considered a failed intubation. Glottic visualization was also evaluated using the Cormack and Lehane classification system and recorded. The difficulty of nasotracheal intubation was assessed with the modified NIDS (Table 1)^24^, a NRS ranging from 0 (easiest) to 10 (most difficult). In addition, the quality of navigation at each stage including the insertion of tube from the nose to oropharynx, the alignment of the tube into the glottic inlet, and the advancement of the tube into the trachea was graded, respectively^15^. The insertion of the tube into the nose to oropharynx was classified as grade 1 (smooth passage), grade 2 (side-to-side rotation), or grade 3 (use of the other nostril due being unduly impinged). The alignment of the tube from the oropharynx into the glottic inlet was classified as grade 1 (smooth or use of manipulation such as right-hand manipulation of the tube and backwards upwards rightward pressure manoeuvre), grade 2 (use of the cuff inflation method) or grade 3 (use of Magill forceps). We used the cuff inflation method when the tube tip failed to align with the glottic inlet by grade 1 manipulation, as follows: Under laryngoscopic view, the anaesthesiologist requested an assistant to inflate the cuff with a syringe to lift the tube tip off the posterior pharyngeal wall until the tube tip was aligned with the glottic inlet; the tube was then advanced. Once the tube tip has passed the glottic inlet, the cuff was deflated, and the tube was advanced into the trachea. The advancement of the tube from glottic inlet into the trachea was scored as grade 1 (a smooth advancement), grade 2 (use of the tube-rotation method), or grade 3 (use of Magill forceps). If manipulation using Magill forceps failed at any stage, the next method was subsequently selected at the discretion of the anaesthesiologist and recorded.

When removing the laryngoscope, epistaxis was scored as grade 1 (no-epistaxis), grade 2 (mild, blood on the tube only), grade 3 (moderate, blood pooling in the pharynx), or grade 4 (severe: blood impedes intubation)^25^. At this point, the study was completed, and anaesthesia was maintained with sevoflurane (end-tidal concentration: 1.5–2.5%) in a 50% oxygen and oxygen-air mixture, and suitable blood pressure and heart rate were monitored and maintained.

### Statistics

The primary evaluation endpoint of this study was the time taken for intubation. Based on the estimated standard deviation from a previous study of 15 s^11^, a sample of 36 patients per group was calculated to provide 80% power to detect a between-group difference of 10 s in the intubation time using an alpha level (2-sided) of 0.05^6,11,26^. Secondary endpoints of this comparative study included NIDS, NRS, the quality of navigation at each stage of tube insertion, and epistaxis. Statistical analysis was performed using IBM SPSS statistics version 21 (IBM Corp., Armonk, New York, USA). All analyses were performed according to the intention-to-treat principle. All data were tested for normality using the Kolmogorov-Smirnov normality test. Data are presented as the mean and standard deviation where normally distributed, or median and interquartile range where non-normally distributed, or as a percentage, as appropriate. Normally distributed data were analysed using a one-way analysis of variance, followed by the Tukey post-hoc analysis if a statistically significant difference was detected. Non-normally distributed data was analysed using the Kruskal-Wallis test, followed by the Mann-Whitney U-test using the Bonferroni adjustment. Categorical variables were analysed using the chi-square test or Fisher’s exact test, followed by Bonferroni adjusted chi-square test or Fisher’s exact test. A P value < 0.05 was considered statistically significant and a P value < 0.017 was considered statistically significant with the Bonferroni adjustment.

## Data Availability

The authors declare that all data supporting the findings of this study are available within the article and its Supplementary Information files, or from the corresponding author upon request.
